# Impact of Sediment Concentration on the Survival of Wastewater-Derived *bla*_CTX-M-15_-Producing *E. coli*, and the Implications for Dispersal into Estuarine Waters

**DOI:** 10.3390/ijerph17207608

**Published:** 2020-10-19

**Authors:** Yasir M. Bashawri, Peter Robins, David M. Cooper, James E. McDonald, Davey L. Jones, A. Prysor Williams

**Affiliations:** 1 General Directorate of Environmental Health, Ministry of Health, Riyadh 12234, Saudi Arabia; ybashawri@moh.gov.sa; 2 School of Natural Sciences, Bangor University, Bangor LL57 2UW, UK; j.mcdonald@bangor.ac.uk (J.E.M.); d.jones@bangor.ac.uk (D.L.G.); 3 School of Ocean Sciences, Bangor University, Bangor LL59 5EG, UK; p.robins@bangor.ac.uk; 4 Centre for Ecology and Hydrology, Environment Centre Wales, Bangor LL57 2UW, UK; cooper@ceh.ac.uk; 5 UWA School of Agriculture and Environment, The University of Western Australia, Crawley 6009, Australia

**Keywords:** bathing waters, extreme weather, pathogens, sewage, microbial pollution, water framework directive

## Abstract

The environmental cycling of antibiotic-resistant *bla*_CTX-M-15_-producing *E. coli* following release from wastewater treatment plants is a major public health concern. This study aimed to (i) assess the impact of sediment concentrations on the rate of their inactivation following release from human wastewater into freshwater, and (ii) simulate their subsequent dispersal to the nearby coastline during a “worst-case” event where heavy rainfall coincided with high spring tide in the Conwy Estuary, North Wales. Freshwater microcosms of low, medium and high turbidity were inoculated with *bla*_CTX-M-15_-producing *E. coli*, then exposed to ultraviolet (UV) radiation. Typical regional wintertime exposure to UV was found to be insufficient to eradicate *E. coli*, and in highly turbid water, many bacteria survived simulated typical regional summertime UV exposure. Modelling results revealed that *bla*_CTX-M-15_-producing *E. coli* concentrations reduced downstream from the discharge source, with ~30% of the source concentration capable of dispersing through the estuary to the coast, taking ~36 h. Offshore, the concentration simulated at key shellfisheries and bathing water sites ranged from 1.4% to 10% of the upstream input, depending on the distance offshore and tidal regime, persisting in the water column for over a week. Our work indicates that the survival of such organisms post-release into freshwater is extended under typical wintertime conditions, which could ultimately have implications for human health.

## 1. Introduction

Antibiotics such as those in the β-lactam (Beta-lactam) group are widely used in clinical and community settings to treat human infections. Frequent bacterial exposure to β-lactam antibiotics, such as penicillin, carbapenem, monobactams and cephalosporins, may cause bacteria to develop the ability to produce so-called Extended-Spectrum β-Lactamase enzymes (ESBLs), which hydrolyse β-lactam antibiotics, making them ineffective in infection control [1,2].

Wastewater treatment plants (WWTPs) represent major point sources for the release of antibiotic-resistant bacteria (ARB) and antibiotic-resistant genes into the environment [3,4,5,6]. Of these, the ESBL *bla*_CTX-M-15_ gene has become highly prevalent, especially in *E. coli*, and can be readily detected at high concentrations in wastewater, freshwater and marine environments [7,8,9]. For example, a study by Bréchet and colleagues (2014) estimated that more than 600 billion ESBL-producing *E. coli* were released daily via treated wastewater into a receiving river in France [10].

When WWTP discharge pathogenic bacteria into river or marine environments, the bacteria face many environmental challenges. These can include changes in water chemistry (e.g., pH, nutrients, salinity, toxins, O_2_), climate (e.g., ultraviolet (UV) radiation, temperature), physical environment (e.g., aggregation) and biological pressures (e.g., competition, predation, viral lysis) [11,12]. Bacterial attachment to suspended sediment particles has been shown to play an important role in the transport of bacteria in the wider aquatic environment [13,14]. In particular, during the high river or tidal flows, high suspended sediment loads in water may encourage greater persistence of ARB, e.g., as a result of higher protection from UV radiation [15], or sediments acting as a reservoir for ARB [16].

The study aimed (i) to assess the impact of the sediment concentration on the survival of *bla*_CTX-M-15_-producing *E. coli* derived from human wastewater under typical seasonal UV exposure in freshwater, and (ii) to use a hydrodynamic model of the Conwy Estuary (North Wales, UK) to simulate the dispersal of *bla*_CTX-M-15_-producing *E. coli* from a discharge point at a WWTP in the river, through the estuary, to coastal shellfisheries and bathing beaches, where there is a heightened risk to human health.

## 2. Materials and Methods

### 2.1. Study Site

The Conwy was chosen for our study due to the available hydrodynamic data from both the estuary and catchment to constrain and validate the model, as described by Robins et al., (2014) [17]. The river Conwy has a drainage basin of 590 km^2^, with annual rainfall between 1200 and 3500 mm, depending largely on altitude. Mean flow in the river is 20 m^3^/s (1965 to 2015), with peak flows exceeding 500 m^3^/s [18]. The estuary is approximately 20 km long, up to 10 m deep, with bed material mainly comprised of silts and medium sands. It is a well-mixed, macro-tidal estuary with a tidal range of 4–6 m [19]. The estuary and nearshore coast is an important area for the cultivation of mussels [20] and a tourist region with several blue-flag bathing beaches. There are three main WWTPs, more than 15 smaller WWTPs, and 35 combined sewage outfalls that discharge into the river network [21]; all potential sources of bacterial contamination [22]. A detailed description of the hydrology and catchment can be found in Emmett et al. (2016) [23].

### 2.2. Sample Collection and Characterisation

Triplicate water samples were collected from 15 cm below the water surface, 200 m downstream of the main WWTP outlet at Llanrwst (53°8′24″ N, 3°48′10″ W), using pre-sterilized 1 L glass bottles. Sediment samples were taken from the same location (at the riverbed) by hand spatula and then transferred into pre-sterilized 2 L glass bottles. Samples were transported at 4 °C to the laboratory within 1–2 h of collection.

Electrical conductivity (EC) and pH were measured in the water samples using a 4850 EC meter (Jenway Ltd., Dunmow, UK) and Hanna precision 209 pH meter (Hanna Instruments, Leighton Buzzard, UK), respectively. Phosphate concentration was determined by the method of Murphy and Riley (1962) [24], and nitrate by Miranda et al., (2001) [25] using a PowerWave XS spectrophotometer (BioTek Instruments Inc., Winooski, VT, USA). Concentrations of total organic carbon (TOC) and total organic nitrogen (TON) were determined using a Multi N/C 2100s CN analyser (Analytik Jena AG, Jena, Germany). Samples of both sediment and water were plated onto Brilliance™ ESBL Agar plates (24 h, 37 °C; Thermo Fisher Scientific Oxoid Ltd., Basingstoke, UK) to test for the presence of ESBL-producing *E. coli* before inoculation [26].

### 2.3. Microcosm Study

#### 2.3.1. Preparation and Inoculation of Turbid Water

The level of turbidity in freshwater typically ranges from 50–500 FTU (Formazin Turbidity Units), depending on turbulence and weather extremes (e.g., storms [27,28]). For this study, three different levels of water turbidity (suspended sediment) were obtained by adding sediment from the study site to water samples to give turbidity levels of 10 FTU (low turbidity; LT), 150 FTU (medium turbidity; MT) and 400 FTU (high turbidity; HT). All samples were prepared in triplicate using the independent water and sediment samples. Sediment concentrations were measured before and after adjustment using a HI-93703 turbidity meter (Hanna Instruments, Leighton Buzzard, UK).

An inoculum was prepared from a fresh overnight culture (Nutrient broth; CM0001, Oxoid Ltd., Basingstoke, UK; 24 h, 37 °C, 150 rev/min) of control strain *bla*_CTX-M-15_-producing *E. coli* (NCTC 13353; PHE Culture Collection, Public Health England, Porton Down, UK). A 10 mL aliquot of inoculum was added to 190 mL of each water sample in a 250 mL sterile glass container, then decimal dilutions of samples were plated onto duplicate Brilliance™ ESBL Agar plates, and colonies enumerated after incubation (37 °C, 24 h). The resulting colony-forming units per milliliter (CFU/mL) provided enumeration of ESBL-producing bacteria at time zero (t_0_) (mean 6.74 × 10^7^ CFU/mL across all turbidity levels).

#### 2.3.2. Artificial Sunlight Exposure

Inoculated water samples were exposed to UV light for up to 8 h in a Suntest XXL+FD solar simulator (Atlas Material Testing Technology Inc., Mount Prospect, IL, USA) containing xenon lamps filtered by daylight filters (wavelength range at 300–400 nm, irradiance level of 40 W/m^2^). Previous work at the study site showed that the average exposure to UV radiation per day at a wavelength of 300–400 nm and UV radiation 40 W/m^2^ would equate to 33.6 kJ/m^2^ in winter and 648 kJ/m^2^ in summer (Kata Farkas, personal communication). Based on these figures, 14 min and 4.5 h in our solar simulator would equate to winter and summer UV exposure, respectively. Samples were stirred slowly (100 rev/min) by a magnetic stirrer to maintain a constant turbidity level. Samples were plated and colonies enumerated as described above before exposure (t_0_), and after 0.5 h (t_0.5_), 1 h (t_1_), 2 h (t_2_), 3 h (t_3_), 4.5 h (t_4.5_), 6 h (t_6_) and 8 h (t_8_).

Where no colonies had grown on the media, an enrichment procedure [11] using Brilliance™ ESBL agar was employed to test for the presence of *E. coli bla*_CTX-M-15_. The detection limit of bacterial growth in conventional media is 1 CFU/mL. Those samples that were negative by conventional plating but positive after enrichment were assigned a value equal to half the limit of detection (0.5 CFU/mL).

#### 2.3.3. Isolation and Confirmation of *E. coli*

As well as using chromogenic agar, further tests were conducted to confirm the colonies as ESBL-producing *E. coli*. Six pure single colonies of presumptive *E. coli* were picked randomly by a sterile 1 μL loop from each time (t_0_, t_0.5_, t_1_, t_2_, t_3_, t_4.5_, t_6_ and t_8_) of UV exposure and spread on nutrient agar plates (Oxoid Ltd., CM0003), then incubated at 37 °C for 24 h. Over the course of the study, 126 presumptive *E. coli* isolates randomly picked from plates were all confirmed as *bla*_CTX-M-15_ *E. coli* by the presence of both the *uidA* and bla*_CTX-M-15_* group 1 genes via single-plex polymerase chain reaction (PCR), using the methods described below.

#### 2.3.4. Detection of the *uidA* Gene through Single-Plex PCR

Presumptive *E. coli* isolates were DNA-extracted by a procedure described by Chandra and Goswami (2014) [29] and Dallenne et al. (2010) [30]. In this procedure, a small amount of a single pure colony of *E. coli* grown on nutrient agar was transferred using 10 μL sterile loop into 200 μL of autoclaved distilled water in an Eppendorf tube and vortexed to get a uniform suspension. The bacterial cells were lysed by heating the suspension at 95 °C for 10 min. The cellular debris was removed by centrifugation at 12,000 rev/min for 5 min. The upper aqueous phase was transferred to a new Eppendorf tube. 

Concentrated DNA samples were diluted to roughly 20–30 ng, and absorbance was measured using a spectrophotometer (NanoDrop 1000 Spectrophotometer, Thermo Fisher Scientific, Basingstoke, UK). Diluted DNA was stored at −20 °C until used as the template DNA. 

The *uidA* gene (that enables β-glucuronidase enzyme production) has been used for identification *E. coli*, using primers described by Bej et al. (1991) [31] (UAL-754-F, AAAACGGCAAGAAAAAGCAG; UAR-900-R, ACGCGTGGTTACAGTCTTGCG; amplicon size 147 bp). Single PCR amplification for the *uidA* gene was performed under the following conditions: initial denaturation step at 95 °C for 3 min; 30 cycles of denaturation at 94 °C for 1 min, annealing at 50 °C for 1 min and extension at 50 °C for 1 min; final elongation at 72 °C for 7 min.

For *uidA*, a 50 μL reaction mixture consisting of 25 μL (1x) BioMix Red (Bioline, London, UK), 1 μL *uidA*-F primer, 1 μL *uidA*-R primer, 21 μL Water Molecular Biology Reagent (W4502, Sigma-Aldrich, Dorset, UK) and 2 μL DNA template was used. PCR-amplified fragments (5 μL aliquots) were separated and run on 2.5% (w/v) agarose gels. Agarose gels were run in 1x-TBE buffer (0.09 M Tris-borate and 0.002 M EDTA, pH 8.0) at 100 V for 25 min. The gels were stained with 10% of SafeView Nucleic Acid (NBS Biologicals, Huntingdon, UK) and visualized under UV light (Molecular Imager^®^ Gel Doc™ XR System, Bio-Rad, Watford, UK). *E. coli* (NCTC 13353) was used as a positive control and distilled water was used as a negative control. All *uidA*-positive DNA were stored at −20 °C for further analysis.

#### 2.3.5. Detection of the *bla_CTX-M-15_* Gene through Single-Plex PCR

Genotypic characterization of ESBL-producers was determined by single-plex PCR assays. The isolates were analysed for the presence of genes targeting *bla*_CTX-M_ group 1 as *bla*_CTX-M-15_ gene and others genes of *bla*_CTX-M_ group 1, using primers described by Dallenne et al., (2010) [30] (CTXM-F, TTAGGAARTGTGCCGCTGYA; CTXM-R, CGATATCGTTGGTGGTRCCAT; amplicon size 688 bp).

PCR reactions were performed as described by Dallenne et al., (2010) [30] in a 50 μL reaction mixture which consisted 25 μL (1x) BioMix Red (Bioline), forward and reverse primers (10 pmol/μL) 0.4 μL CTXM1-F primer, 0.2 μL CTXM1-R primer, Water Molecular Biology Reagent (W4502, Sigma-Aldrich, Dorset, UK) and to 1 μL (20–30 ng) of template DNA.

The amplification protocol of *bla*_CTX-M_ group 1 gene was carried out as described by Dallenne et al., (2010) [30]. After PCR amplification, amplified products were visualized using 2.0% agarose gel electrophoresis. The gel consisted of 1x-TBE buffer (0.09 M Tris-borate, and 0.002 M EDTA, pH 8.0) and stained using 10% of Safe View Nucleic Acid (NBS Biologicals). Products were run at 100 V for 30 min and visualized under UV light (Molecular Imager^®^ Gel Doc™ XR System, Bio-Rad). *E. coli* (NCTC 13353) was used as a positive control for *bla*_CTX-M-15_ (group 1), and *E. coli* (NCTC 10418) as an ESBL-negative control.

#### 2.3.6. Statistical Analysis

Changes in *E. coli* numbers (CFU/mL) at the different sampling times (t_0_, t_0.5_, t_1_, t_2_, t_3_, t_4.5_, t_6_ and t_8_) for each turbidity level were evaluated with SPSS Statistics 22 (IBM, New York, NY, USA) and Excel 2013 (Microsoft, Albuquerque NM, USA). Because of non-normal distribution, significant differences were identified using non-parametric tests: Kruskal–Wallis one-way ANOVA to ascertain differences in survival between waters of different turbidity, and Mann–Whitney to ascertain changes in numbers over time within the same level of turbidity. *p*-values < 0.05 were considered statistically significant.

### 2.4. Model Development

A vertically-averaged hydrodynamic model (Telemac V7.2; www.opentelemac.org; [32]) was applied to the estuary and surrounding coast. The model was constrained with the most recent available bathymetry data for the region, mapped onto an unstructured mesh that was optimised to adequately resolve estuarine and coastal currents (see Robins et al., 2019 [33] for further details of the model parameterization). Tidal forcing was derived from the Oregon State University TOPEX/Poseidon Global Inverse Solution tidal model (TPXO) global database [34] and the model was spun up to create a steady-state salinity balance under low river flow (Q99 of 1 m^3^/s) conditions. Comprehensive validation procedures have previously been conducted for hydrodynamics and salinity intrusion [17] which test the suitability of the model in the depth-averaged mode for application to the Conwy.

Based on the findings of the microcosm study, where survival of *bla*_CTX-M-15_ -producing *E. coli* was enhanced under conditions of high turbidity, we simulated an extreme, but possible, combination event of extreme river flows combined with spring tides lasting two weeks (what could be envisaged as a “worst-case” set of scenarios), during which time the contamination event was modelled as a continuous input. Such scenarios might become increasingly common under climate change predictions, with elevated patterns of high-intensity rainfall that would lead to re-suspension of sediments in water bodies. Under this scenario, realistic high river flows and spring tides occurred in such a way as to produce a possible maximum export seaward of *bla*_CTX-M-15_ -producing *E. coli* in water of high turbidity.

**Figure 1:**
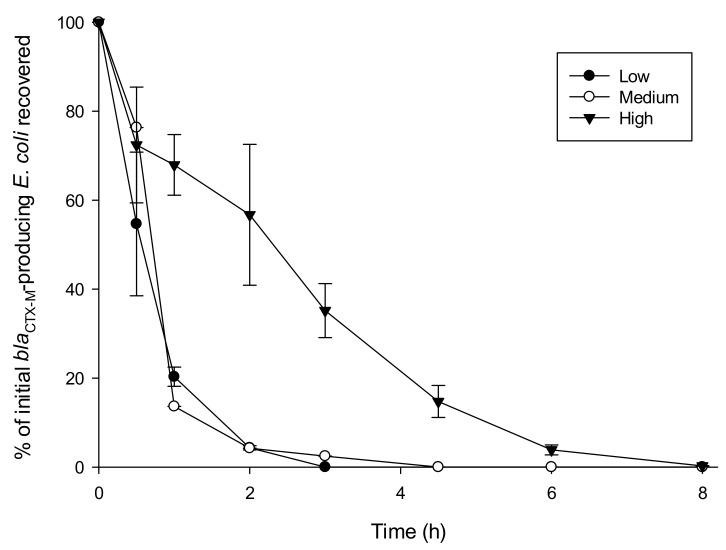
Percentage of initial (*t*_0_) *bla*_CTX-M-15_-producing *E. coli* recovered in the freshwater of different turbidity levels (low, medium or high) over time, following exposure to UV irradiation. Based at the irradiance level of 40 W/m^2^, the equivalent UV dose exposure in winter would be after 14 min, and in summer would be after 4.5 h.

To simulate the decay of *bla*_CTX-M-15_ -producing *E. coli*, the die-off rate t_90_ was calculated to be 2 h and 15 min, based on the microcosm study of high turbidity (interpolated between points; Figure 1). Within the model, we simulated concentrations, C, with a die-off function following Equation (1):

(1) $$C=C_{0}e^{-kt}$$

where $C_{0}$ is the measured concentration at time (*t*) zero and $k=-ln\left(0.1\right)/t_{90}$.

Using gauged river flow measurements for the Conwy (1980 to 2015), we isolated the maximum freshwater inflow to the estuary over a 7-day period, which comprised six high-flow events within six days, with three events of peak magnitudes of 350, 470 and 450 m^3^/s, which occurred between 30 January–13 February 2004. These flows were used in conjunction with a constant input of *bla*_CTX-M-15_ -producing *E. coli* from Llanrwst STP to create a 14-day time series of the river concentration boundary forcing at the tidal limit. For this, the input concentrations were multiplied by an estimated flux of water from the WWTP to give an estimated river concentration. At the offshore boundary of the model, the simulation was constrained with the largest annual astronomical tides (tidal range exceeding 6 m), aligning the tidal phase in such a way so that peak river flows coincided with peak ebb tidal flow and maximum concentrations were dispersed offshore.

## 3. Results

### 3.1. Characterization of Samples

The physiochemical and microbiological characteristics of the water used in the study are displayed in Table 1. As with the water sample, no *E. coli* were recovered in the sediment prior to inoculation.

### 3.2. Influence of Turbidity on Survival of bla_CTX-M-15_-Producing E. coli

In water of low turbidity, numbers of *E. coli* were reduced to below the detection limit at T_8_ but were positive by enrichment. The UV radiation experienced during simulated winter sunlight exposure did not eliminate bla_CTX-M-15_-producing *E. coli* at any turbidity level. However, simulated summer exposure to UV significantly reduced numbers of bla_CTX-M-15_-producing *E. coli*, by 99.99% in water of low turbidity, 99.98% in water of medium turbidity, and 85.24% in water of high turbidity (Figure 1). Overall, mean counts of bla_CTX-M-15_-producing *E. coli* between low, medium and high turbidity were significantly different, with greater survival at higher turbidity (Kruskal–Wallis one-way ANOVA, *p* < 0.05; data not shown).

To estimate the decay parameter, k, under different levels of turbidity, we first scaled the counts for the three treatments by taking their ratio to the mean of the counts at t_0_. Using the R routine lm, we then fitted a straight line through the natural logarithms of these scaled values, forcing the line through zero (equivalently log_e_1). Fitting in this manner is one way of accounting for the greater variability in data at higher counts. Each of the three fitted lines can then be expressed in Equation (2) as

log_e_ (y/y_0_) = −k × *t* (2)

or equivalently in Equation (3):

y = y_0_ exp(−k*t*) (3)

where y represents the raw counts, y_0_ is the mean of the measured initial counts, *t* is time in hours from the start of the experiment, and k is a decay parameter. The fitted k-values and their standard errors are shown in Table 2. These reiterate the marked increase in the rate of die-off in waters of lower turbidity.

### 3.3. Dispersal of bla_CTX-M-15_-Producing E. coli within the Receiving Estuary

**Figure 2:**
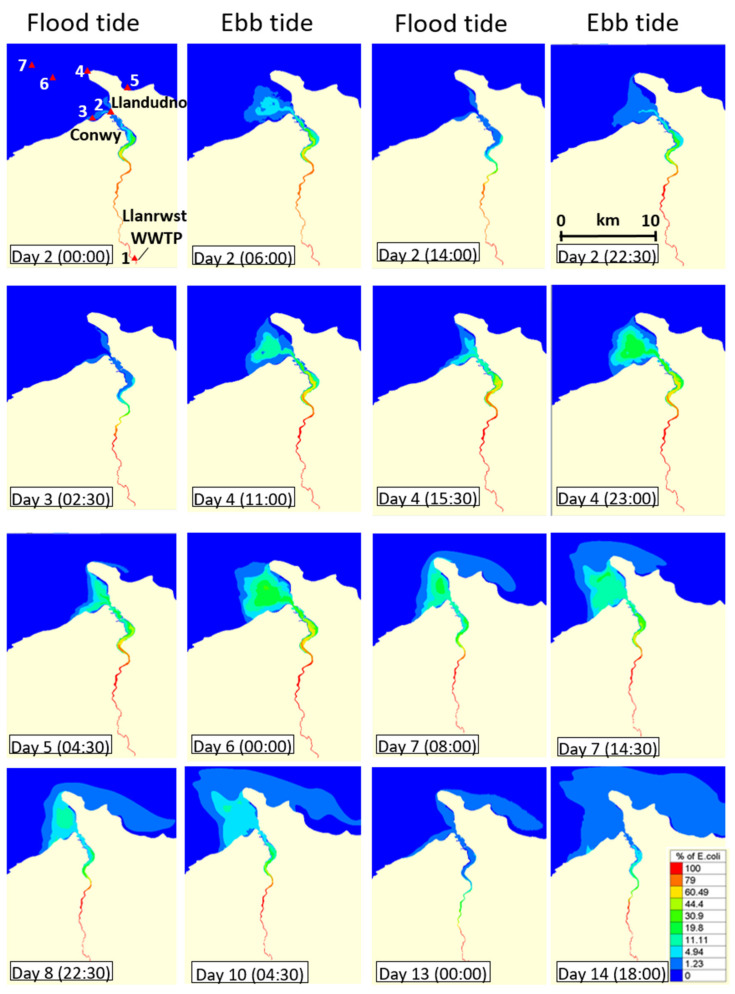
Maps of the Conwy Estuary and surrounding North Wales’ coast showing simulated dispersal of percentage concentration of *bla*_CTX-M-15_-producing *E. coli* from a continuous point source (river head) over 14 days during a “worst-case” event where heavy rainfall coincided with high spring tides. The simulation and contamination started at day 0 and results shown from day 2 onwards. The seven locations, Sites 1–7, are: 1 = discharge effluent, 2 = estuary mouth, 3 = Conwy Morfa Beach, 4 = Llandudno Head, 5 = Llandudno Beach, 6 and 7 = offshore in the Irish Sea.

The above results strongly indicate that water of high turbidity can help extend the survival of *bla*_CTX-M-15_-producing *E. coli* released from a WWTP. Based on the rate of die-off seen in the microcosms of highly turbid waters, we simulated an extreme release of *bla*_CTX-M-15_-producing *E. coli* from a WWTP in the river Conwy, after a period of heavy rainfall and maximum river flows (and turbidity)—a so-called “worst-case” culmination of scenarios, as described earlier. Strong flows can, of course, dilute *E. coli* concentrations, although our results show percentage concentrations relative to the river loading, and were designed to simulate spatial and temporal patterns of dispersal, based on extrapolating the rate of die-off seen in highly turbid water during preliminary labwork. Under the simulations, the *bla*_CTX-M-15_-producing *E. coli* mixed with the seawater as it dispersed downstream to the estuary, reducing the concentration. For instance, in the lower estuary more than 10 km from the source, the *bla*_CTX-M-15_-producing *E. coli* concentration was reduced by more than 50% (Figure 2). Only during the ebbing tide were *bla*_CTX-M-15_-producing *E. coli* exported out of the estuary, although a small proportion re-entered the estuary during the subsequent flood tide.

**Figure 3:**
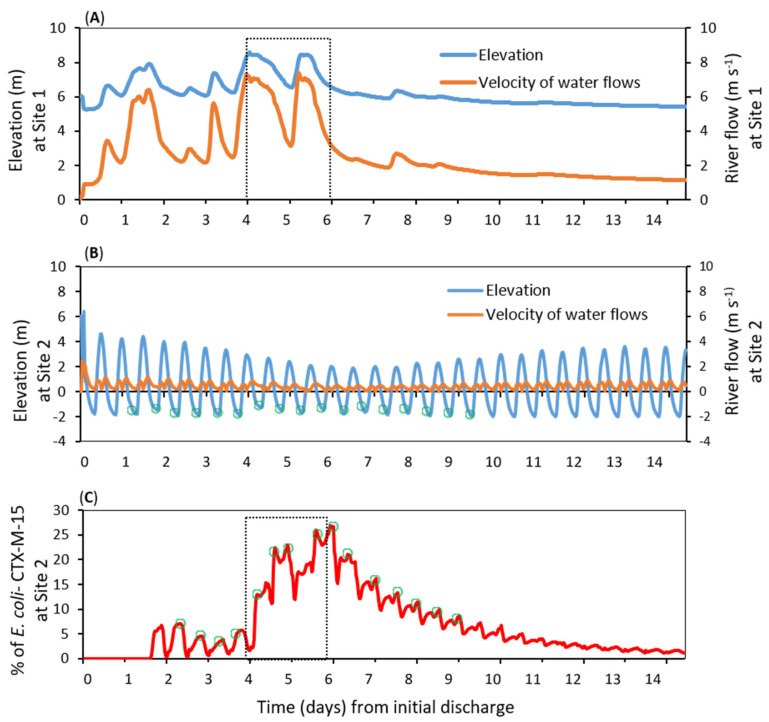
Simulation of water level (m) and river flow (m/s) at (**A**) the wastewater discharge point (Site 1) and (**B**) the estuary mouth (Site 2); (**C**) *bla*_CTX-M-15_-producing *E. coli* concentration at the estuary mouth. Dashed squares denote the storm period, with the highest flows and *E. coli* loadings. Green cycles show peaks in concentration during ebb tides.

Seven locations were selected to visualise the fate of *E. coli* dispersal during the 14-d model simulation (Figure 2). Two locations were within the estuary—the discharge source and the estuary mouth. Three locations were in inshore waters and two locations further offshore (>5 km from the estuary). These coastal locations represented actual commercial shellfisheries and tourist beaches. Under the simulated “worst-case” scenario of highly turbid water, peak simulated percentage concentrations of *bla*_CTX-M-15_-producing *E. coli* at the downstream sites were 27% (Site 2), 10% (Site 4), 8% (Site 6), 4% (Site 7), 4% (Site 3) and 1% (Site 5). From the WWTP source, high velocity simulated river flows quickly advected the *E. coli* downstream through the 20 km estuary to the mouth (Site 2) in approximately 36 h (Figure 3A,C). Peak river flows of 7.2 m/s during the storm event (days 4–6) resulted in simulated peak concentrations at the estuary mouth approximately 12 h later (Figure 3A,C). After the storm event had passed (after day 6), the reduced river flows led to reduced simulated concentrations of *E. coli* at the estuary mouth; concentrations reducing from 27% to less than 5% after approximately 5 days and also modulated by tidal motions (Figure 3B,C).

**Figure 4:**
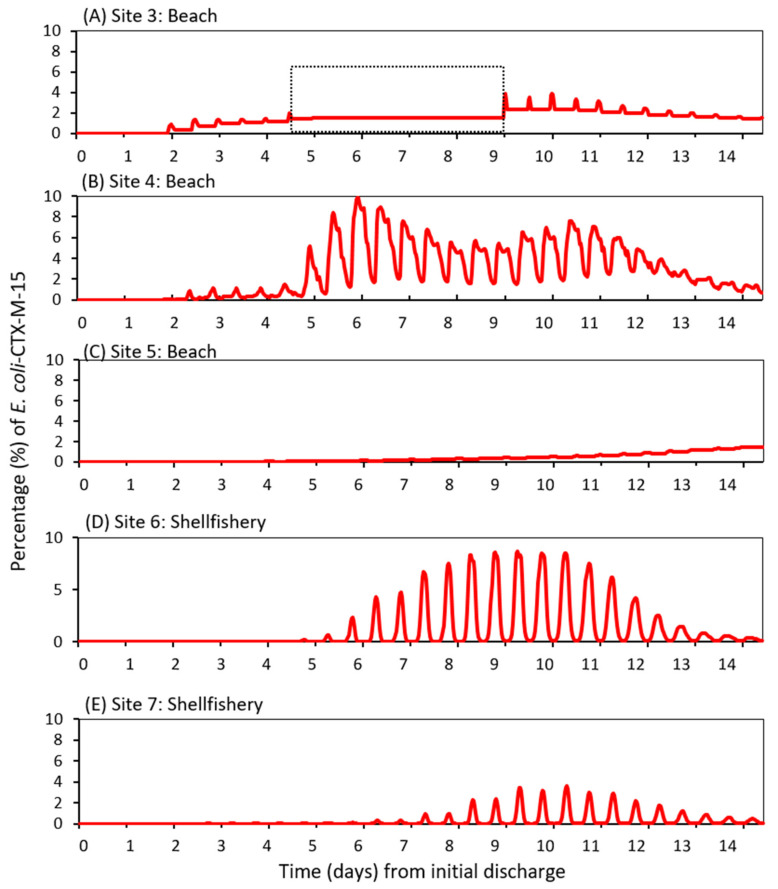
Simulated *bla*_CTX-M-15_-producing *E. coli* at Sites 3–7 (**A**–**E**). The dashed square in (**A**) denotes a period of neap tides when Site 3 was dry and the simulated *E. coli* concentrations were unchanged.

Under the model assumptions, *E. coli* took approximately two days to reach the beaches flanking the estuary, whereas it took approximately one week to reach beaches further afield (Llandudno 5 km away) (Figure 4A,C). An interesting dispersal pattern was simulated at Site 4 (Figure 4B): relatively large concentrations of *bla*_CTX-M-15_-producing *E. coli* (peaking at 10% on day 6) were simulated following the storm event and subsequent ebb tide. The source concentration reduced hereafter, whereas the simulated concentration at Site 4 remained high and then increased again (second peak of 8% on day 10) because of the modelled effect of a strengthening tide approaching the next spring phase. Hence, while near-source locations in the river and estuary would be at heightened risk shortly after a high loading event, the risk for locations further offshore would be contingent on both high loading events and the tidal regime. In other words, the risk offshore might be heightened for longer periods, albeit at lower levels of risk compared with near-source sites.

Simulated *bla*_CTX-M-15_-producing *E. coli* reached the shellfisheries Sites 6 and 7 after ~5 days (114 h and 123 h, respectively) (Figure 3D,E). Hence, we predict a lag in the timings of peak concentration from that at the source in the river to that further downstream and offshore. Model results also suggest that tidal patterns were an important factor in dispersing concentrations of *bla*_CTX-M-15_-producing *E. coli* in offshore waters, and could lead to the second period of high concentrations and heightened risk.

## 4. Discussion

The discharge of ARB and their genes from WWTPs have been shown to negatively impact water quality within downstream coastal environments [35]. Several studies have also found that river sediments can be a source of ESBL-producing *E. coli* and their genes (e.g., [16,36]). This study adds to this knowledge as it elucidates how turbidity levels in freshwater affect the survival of *bla*_CTX-M-15_-producing *E. coli* under exposure to different doses of UV between seasons. We then applied the findings to a validated hydrodynamic model to predict how stormriver flows containing *bla*_CTX-M-15_-producing *E. coli* originating from a WWTP source can be transported within the estuary and surrounding coast. Small hydrological systems like the Conwy are economically important and ecologically productive regions that outnumber the larger systems within the UK, and their collective contribution in the delivery of freshwater, nutrients and viruses to the shelf seas is large [37]. By simulating a “worst-case” load export scenario during a combined event of storm river flow and large spring tides, we estimate the spatial and temporal risk of exposure to *E. coli*. Hence, the timings of heightened risk due to storm events can be determined, e.g., for key locations such as shellfisheries and bathing beaches.

Our work showed that suspended sediment concentrations influence the survival of *bla*_CTX-M-15_-producing *E. coli* in waters exposed to UV. In particular, survival was enhanced under higher turbidity that could occur due to a storm event (Figure 1, Table 2). These observations are concordant with those of Perkins et al., (2016) [38], which demonstrated attachment of bacteria in highly turbid water can contribute to enhanced survival due to a combination of reduced penetration of UV and that cells are offered a degree of physical protection (shielding) from UV. At lower turbidity, *E. coli* were exposed to higher levels of UV radiation, which probably led to irreparable damage to different cellular components, causing membrane dysfunction [39,40]. Further work is needed to investigate non-culturable bacterial pathogens and activity over a longer period of UV exposure using advance detection techniques such as quantitative polymerase chain reaction (qPCR). Such a culture-independent method would also help ascertain whether *bla*_CTX-M-15_ genes remain viable even if the *E. coli* have been eliminated. It would also be of interest to model how transport of these bacteria would differ in waters of lower turbidity, although as stated earlier, the intention was to focus on ‘worst-case’ scenarios, where contamination events are most likely.

We found that the simulated wintertime UV exposure had little impact on the survival of *bla*_CTX-M-15_-producing *E. coli* in all levels of water turbidity in freshwater. However, during simulated summertime, a significant reduction in survival of *bla*_CTX-M-15_-producing *E. coli* was seen in all levels of water turbidity. This finding is consistent with the findings of others who have reviewed literature and report that the survival of faecal coliforms in sediment-water was higher in winter than summer [41]. By the time the *E. coli* reach the estuary, strong tidal flushing places coastal waters at-risk of contamination, even during neap tides and low river flows [17] (Robins et al., 2014). Saline water would likely impact on survival, although simulations in the Conwy by Robins et al., (2019) [33] indicate that dispersion of viruses is controlled to a greater extent by the hydrodynamics than by different die-off rates, although bacteria have been characterized to have higher decay rates than viruses under sunlit conditions [42] (Wait and Sobsey 2001) and therefore this should be quantified in future studies.

We are not aware of any previous study on the impact of natural UV exposure (either simulated or in vivo) on the survival of CTX-M-producing *E. coli*. The focus of previous work has been the effect of the dose of UV received by such bacteria during wastewater treatment regimes (e.g., [43]). Such work is useful, although the UV intensity/dose received would be considerably greater than that received through natural exposure, making comparisons with our results difficult. Further, many smaller WWTP do not have a UV stage, therefore work on the susceptibility of such bacteria under more natural UV exposure (experienced post-release from a WWTP) is needed. Other studies have looked at the survival of *E. coli* in waters subject to natural (sunlight) UV. We saw that the difference in survival in waters of medium and high turbidity was less pronounced than between waters of low and medium turbidity (Figure 1, Table 2). This could imply that there is a threshold of turbidity beyond which the effects of UV exposure is less pronounced—this could be explored in further work. Muela et al. (2000) [44] measured the survival of non-CTX-M-producing *E. coli* in sterile river water during exposure to UV-A, and found a 10-fold decrease in numbers after only 24 h. The intensity and wavelengths/spectrum of UV exposure differed between our study and that of Muela et al. (2000) [44], making direct comparisons difficult. Sinton et al. (2007) [45] found that *E. coli* was more resilient to sunlight exposure than *Campylobacter jejuni* and *Salmonella enterica*. There is also some evidence that CTX-M-producing *E. coli* may display enhanced survivability over generic strains of *E. coli* under a range of environmental conditions [46,47], although inter-strain variation in survival should always be borne in mind [12,45]. In the present study, bacterial death might have been accelerated due to the stress that *E. coli* were exposed to after growing in a rich medium at 37 °C, then subsequently transferred into a nutrient-poor, colder environment. Repeating the study using sterile, as well as unsterilized, samples would indicate the relative importance of microbial interactions compared with sediment and UV effects. Collectively, whilst all such work aids our understanding of *E. coli* decay under UV exposure, we reiterate the danger of extrapolating the findings of a single experiment to predict persistence under particular environmental conditions.

Our results showed a positive relationship between river flow and *bla*_CTX-M-15_-producing *E. coli* concentration at the estuary mouth, suggesting that the risk to water quality within the estuary is strongly contingent on river flow, hence, rainfall. In the lower estuary and offshore, the influence of tidal movements becomes the driving force on dispersal. This key region contains commercial mussel beds, blue flag beaches and recreational waters, and there was a complex relationship between the concentration of *E. coli* in the water and the timings of the previous storm river flow and the current tidal state. Firstly, the concentration generally reduced and the time lag increased with distance from the source. Secondly, the concentration peaked during ebb tide and spring tides. This complex relationship could generate an initial peak in concentration due to the peak river flow, but then a second peak due to the spring tide. In effect, the period of heightened water quality risk was lengthened in this region. Whilst further studies are required to characterize the seasonal or inter-annual variability, our results demonstrate the changing controls on water quality risk at different points within a coastal system. We expect our results to hold for similar catchment-estuary typologies, but not for contrasting systems of different size and behaviour/phasing of the drivers, and further work is required to understand how a range of estuary typologies are exposed to public health risks [18].

Our simulations assume that after *bla*_CTX-M-15_-producing *E. coli* are released into the water environment, numbers reduce gradually through the estuary to the coast, in accordance with our t_90_ value. We chose to study the impacts of UV and turbidity on the survival of *E. coli* in freshwater; however, we acknowledge that there are other that could affect survival (e.g., biotic factors, water chemistry, sediment type, water temperature), and that the results of microcosms should be extrapolated to estuary-scale with caution. Such factors could affect the t_90_ value, which would in turn influence modelling outputs. Nevertheless, the work helps illustrate the potential for survival and transmission of *bla*_CTX-M-15_-producing *E. coli* released from a WWTP into a well-studied catchment under a scenario where river water suspended sediment levels are high. The approach used could help improve water quality management strategies.

## 5. Conclusions

Wastewater treatment plants are a known source of antibiotic-resistant *bla*_CTX-M-15_-producing *E. coli*, which is a major public health concern. However, the fate of such bacteria following release into freshwaters, and ultimately coastal waters is dependent on several factors. We found that water of greater turbidity level, such as incurred during storm events, offer protection to *bla*_CTX-M-15_-producing *E. coli* against major abiotic stress—UV exposure. Using models, we predicted how such bacteria are transported under a “worst-case” scenario, where heavy rainfall (and high turbidity) coincided with high spring tide in the Conwy Estuary, North Wales. Results indicated that key shellfisheries and bathing sites were at risk of contamination, although the risk attenuated over time. Our results indicate that the survival of such organisms post-release into freshwater is expected under wintertime conditions, which may have implications for human health.

## Figures and Tables

**Table 1:** Chemical and microbiological characteristics of the water.

Parameter	Mean ± Standard Error of Mean (SEM)
pH	7.41 ± 0.09
Electrical conductivity (mS/cm)	0.10 ± 0.01
Total organic carbon (mg/L)	5.92 ± 1.10
Total organic nitrogen (mg/L)	1.42 ± 0.19
Nitrate (mg/L)	0.89 ± 0.01
Phosphate (mg/L)	0.02 ± 0.01
ESBL *-producing *E. coli*	None detected

* Extended-Spectrum β-Lactamase.

**Table 2:** Fitted k-values for *bla*_CTX-M-15_-producing *E. coli* decay in waters of different turbidity levels over time, following exposure to UV irradiation.

Turbidity Level	k-Value	SEM	R^2^
Low	2.80	0.09	0.88
Medium	1.95	0.06	0.96
High	0.62	0.03	0.96
